# “Move or Not to Move”—Red Deer Stags Movement Activity during the Rut

**DOI:** 10.3390/ani12050591

**Published:** 2022-02-25

**Authors:** Erika Csányi, Tamás Tari, Sándor Németh, Gyula Sándor

**Affiliations:** 1 Institute of Wildlife Biology and Management, Faculty of Forestry, University of Sopron, H-9400 Sopron, Hungary; tari.tamas@uni-sopron.hu (T.T.); sandor.gyula@uni-sopron.hu (G.S.); 2 Fauna South-Transdanubian Hunting Association, H-7561 Nagybajom, Hungary; sandornemeth18@gmail.com

**Keywords:** mating behaviour, red deer, conception, activity, GPS telemetry

## Abstract

**Simple Summary:**

The spatial and temporal structure of the movement of animals is based on behavioural decisions that promote evolutionary success. One of these behavioural decisions is mating strategy. In this paper, we examined the relationship between the movement activity of red deer stags and the fertile period of hinds. We hypothesized that the oestrus of hinds significantly affected the daily activity of stags. We determined the oestrus period of hinds, which was compared with the movement data of the stags determined by GPS telemetry. The combination of GPS telemetry data and oestrus data for red deer has never been used in parallel to demonstrate how the reproductive activity of females affects the spatial behaviour of males in red deer. Knowledge of the movement behaviour of red deer during the mating season is important information in both nature conservation and game management.

**Abstract:**

Detailed animal movement analysis can help understand spatial population processes as the ultimate consequences of individual behaviour and ecological impacts. The mating strategy of mammalian herbivores is adapted to the distribution of females; thus, it is important to observe the activity of animals during a rut. In this paper, we used a new approach to examine the relationship between red deer stags’ movement activity and the fertile period of hinds. We presumed a relationship between stags’ daily activity changes and the period when hinds are in heat. We determined temporal conception trends, including the first and last conception dates in the examined population and the conception peak. In the same period, i.e., in the interval of major ecological significance when mating takes place, the activity of stags was analysed by GPS telemetry. The data collected in the examined period indicate that 60% of the hinds were conceived between 31 August and 19 September. We demonstrated that conception rates differed significantly between the first and second half of the rutting period. At the beginning of the reproductive cycle, the high number of hinds on heat (oestrus synchrony) increased the activity of stags (harem herding) compared to the pre-rutting period. As the mating season progressed, the movement activity of stags increased more (searching behaviour), induced by the decreasing number of fertile hinds. Therefore, we found that the oestrus of hinds significantly influenced the movement activity of stags in red deer.

## 1. Introduction

Red deer (*Cervus elaphus*) is one of the most widespread large herbivore species in Europe. Populations and distribution areas have significantly increased in the past decades [1,2]. The most important factors of population dynamics include the reproductive traits of females and the factors influencing these [3]. The mating strategies observed for certain taxonomical groups of mammals depend on females’ temporal and spatial distribution in oestrus [4,5]. In many cases, it is not obvious whether the spatial distribution of females in the mating season is influenced by ecological factors or, by means unknown, by the distribution of males [6].

The number of studies related to movement ecology increased in the past decade, establishing the opportunity to use information on individual movement patterns (home range and its characteristics, daily activity, and habitat use) to promote the understanding of the behaviour of species while also serving the purpose of planning wildlife management and environmental protection [7]. The spatial and temporal structure of the movement of animals is based on behavioural decisions that promote evolutionary success while the animals respond to numerous physical, biological, and environmental stimuli [8,9,10]. With a crucial evolutional relevance, one of these behavioural decisions is the mating strategy.

Among cervids, as a highly polygynous species, red deer males are capital breeders, as their energy for reproduction comes from their accumulated reserves in the previous spring and summer period with a huge body mass loss of adult males during the rut (on average: 19,5%) [11,12]. They are characterized by harem defence polygyny. Stags compete for and defend hinds and mate with those in the harem during heat [13]. Regarding competing stags, dominance depends on body size [8], antler size [14], and age [15] as well as population-related parameters such as the concentration of hinds, sex ratio, and age distribution [16,17]. The mating strategy of stags is influenced by the spatial and temporal distribution of hinds in oestrus, which is affected by the distribution of food sources [4,5,18]. In the case of stags, successful reproduction also depends on the outcome of competition with others [19,20].

The mating season of free-range red deer, known as the rut, lasts hardly more than a month, and most hinds are fertilised in the first half of this period [21,22]. Fertilisation success depends on hind condition [23,24]. In some cases, it is also influenced by the age of the hind or its position in the social hierarchy [3]. Parturition time is a key factor in the survival of calves. The oestrus of hinds within a herd is synchronized [25]. Most female cervids are polyestrous (e.g., roe deer is mono-oestrus), and nonpregnant animals can exhibit either continuous estrous cycles (e.g., some tropical species) or, more typically, alternating periods of oestrous cyclicity and anestrus. It is important to consider that continuous cervid oestrous cyclicity is not a normal phenomenon, particularly those with high seasonal breeding activities. Return to oestrus/ovulation following a conception failure increases the likelihood of establishing a subsequent pregnancy. Evolutionary pressures for maintaining a well-synchronized calving season at a reasonable time of year are quite strong. The consequences of conceiving and calving later in the season, particularly in harsh habitats, can cause neonatal deaths. As a result, many seasonally breeding cervids exhibit high conception rates during their first oestrus (85% in red deer and fallow deer). They then terminate further ovulatory activity for the season [26]. In areas with a higher population density, the ovulation of hinds is more synchronized than in those with a lower population density [27].

Many papers proved seasonal differences in the activity of red deer. The range of movement is smaller in the summer than in the winter [28,29,30]. Adults are characterized by a stable range of movement. The daily activity of red deer shows peaks right after sunrise and around sunset [30,31] 

Like males of other ungulate species [32], stags live separately from hinds outside the breeding season. At the start of the breeding season, stags often extend their normal range of movement [4,28] or migrate larger distances [33] to look for females. Red deer are not territorial [34]. During the mating season, the daily activity of stags increases [35]. One of the reasons for this is the herding behaviour of stags that might significantly limit the movement of hinds in oestrus. The movement of adult stags during mating is also influenced by competition and the search for hinds on heat [19,30,36]. Furthermore, stags should be available for hinds in a relatively short period (24–48 h) when they are fertile [21,28,37]. 

This paper aimed to study how the reproductive activity of females affect the spatial behaviour of males in red deer. We combined two science-based methods, which have never been used in parallel, biological, and behavioural traits in both sexes of this species, i.e., reproduction/fertility of females (hinds) and spatial behaviour of males (stags). 

## 2. Materials and Methods

### 2.1. Study Area

Our study was performed in the Boronka Protected Landscape Area of Somogy County in the southwestern part of Hungary (centre: 46.452332 N; 17.489244 E) between 1 August 2019 and 29 February 2020. The study area is a plain that covers almost 11,000 hectares. As for its vegetation, 75% of the area is covered by temperate continental mixed forests. About 15% of the area is intensively cultivated, and the most important arable crops are maize, sunflower, wheat, barley, oat, and fodder radish. Protected Natura 2000 natural grasslands cover 10% of the area. Native large mammals include red deer, roe deer (*Capreolus capreolus*), wild boar (*Sus scrofa*), red fox (*Vulpes vulpes*), and golden jackal (*Canis aureus*) and, as a non-native species, the fallow deer (*Dama dama*) is observed [38]. 

Regarding wildlife management, the most important game species is red deer. The population density of red deer is high in the area. Population estimation was carried out at the end of February, after the end of the hunting season using static census methods. The estimated number of red deer was 1000, with a sex ratio of 1:3 regarding stags and hinds. In the past 5 years, the average cull of red deer was 480.

### 2.2. Capturing

Manually and electrically operated drop nets were used to capture 4 red deer stags in 2018 and 2019. The estimated age of the stags was above 7 years. Animals captured in the nets were secured and injected with a uniform intramuscular dose of 150 g/body weight kg of 10 mg/mL Cepesedan® (CP-Pharma Handels GmbH, Ostlandring 13, 31303 Burgdorf, Germany). Captured animals were fitted with Vertex Lite collars manufactured by Vectronic Aerospace GmbH. Collars were set to signal the position of animals once an hour (corresponding to 24 positions a day). Recorded data were transmitted using the Iridium satellite constellation.

### 2.3. GPS Data Processing

Processed data included a total of 8769 GPS positions. The R software (R Foundation for Statistical Computing, Vienna, Austria) performed basic operations (filtering, classification, and analysis) [39]. GIS software TopoLynx-topoXmap (TopoLynx Kft., Kőszeg, Hungary) was used for map visualisation. To establish movement ranges for individual animals, the Kernel 90 (KHR90) method was applied [40]. Movement ranges were calculated by R adehabitatHR [41]. Ranges were visualised by adaptive kernel density estimation with an ad hoc smoothing parameter [42]. The smoothing parameter value was adjusted according to the characteristics of the examined data series [43,44]. Hence, ad hoc = 0.7 x href was used, a parameter successfully applied earlier when determining the movement range of deer species [45]. When calculating locomotor activity, the distances of the positions measured in meters along the polyline created by connecting subsequent positions (taken each hour) were used [46]. Polyline was created using ArcView 3.2 Esri (Environmental Systems Research Institute Redlands, California, U.S.) Animal Movement Analyst Extension [47]. Daily activity was calculated by means of first establishing the time of day with the lowest activity; it was 12:00 (noon). Using this as a reference point, daily activity was defined as the sum of the distances between the positions taken from 12:00 (noon) on a given day until 11:00 the next day. In this manner, the activity of individuals leaving their resting place and returning to it could be described.

### 2.4. Establishing the Time of Conception

As a part of our study, we wished to estimate the time of conception and understand gestation-related temporal trends relying on the weight of embryos. 

In placental mammals, there is a linear relationship between the cube root of the weight of the embryo at any given time and the interval between this time point and the date of conception. This relationship applies to the entire gestation period until parturition [48]. This relationship was also demonstrated for several deer species, including wapiti (*Cervus canadensis*) [49,50], fallow deer [51], sika deer (*Cervus nippon yesoensis*) [52], and red deer [50,53,54]. To estimate the pace of development, the gestation period of red deer was taken from the scientific references as 235 days according to the most often cited data. [53,55,56,57]. Relying on this, the specific conception time constant for the species may be calculated (*t_0_* = gestation period × 0.2). To apply the formula by Huggett and Widdas [48], the species-specific growth rate is also needed (a). If we know the weight at birth and the gestation period as well as the conception time constant, the slope of the resulting curve (x coefficient) will provide the growth rate. 

To calculate the species-specific growth rate (a), data collected in Germany by Thomé [53] were used, as the related habitat characteristics resemble Hungarian conditions the most. Hence, the average birth weight was taken as 9.5 kg. The growth rate (a) for this value is 0.113; this is used for all examined embryos as a constant. Relying on these results and embryo weights measured by ourselves, we used the formula below to calculate the number of days since conception [48]:

*T* = (*W1*/*3* / *a*) + *t_0_* (1)

where *T*—number of days since conception; *W*—embryo weight by a growth rate of 0.113; *T_0_*—conception time constant 235 × 0.2 = 47.

According to the above and considering the date of culling the hind, the age of the embryo may be readily calculated, and the date of conception is estimated. During the study period (October 2019–February 2020), 89 red deer hinds with a measurable embryos weight were involved in the data processing. Red deer females were harvested during routine hunts under the quota by the Hungarian authority (2019/2020). The hinds were brought to the laboratory within 2 h from culling, and the embryos were measured immediately. 

### 2.5. Analysis

As a first step, the number of conceptions and activity data was assigned to dates. Days were numbered, starting with 1 August as D_213_. In the case of conceptions, we calculated this with daily and cumulated data (in %). Regarding daily activity, the sum of distances covered daily was taken as being explained under GPS data processing. To smooth the data series, 7-day moving averages were used. For comparisons, the *F*-test was used both for conception and daily activity data. Relying on the results on daily activity, 3 main periods were identified until 29 October (D_303_): Before Rut (D_213-242_, 1 August–30 August), Rut (D_213-242_, 31 August–19 September), and After Rut (D_243-302_, 30 September–29 October). Activity data series were then divided into 5-day sections (quints) with individual activity data. Their names were given by the numbers of their starting and closing days. These 5-day sections were used for the purpose of Principal Coordinates Analysis (PCoA). Accordingly, the Rut period was divided into two halves: the First Half of Rut (D_243-252_, 31 August–9 September) and the Second Half of Rut (D_253-262_, 10 September–19 September). Therefore, the following 4 main periods were analysed, and the following abbreviations were used in the text: Before Rut—BR; First Half of Rut—FHR; Second Half of Rut—SHR; After Rut—AR. Activity and mobility range (KHR90) values in the main periods were analysed by variance analysis (One-way ANOVA) and Tukey’s HSD. A conception index was calculated for individual main periods relying on daily conception data and the duration of the periods. This index is about the number of conceptions in the given main period averaged over days. For comparison, the Chi-squared (χ^2^) test was used. Statistical tests were performed using the software package Past3 [58].

## 3. Results

**Figure 1:**
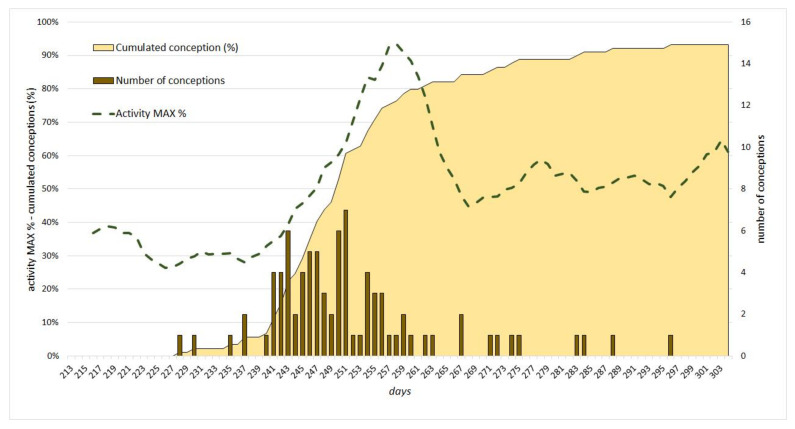
The number of conceptions of red deer hinds and activity trends of stags in the study period. The maximum activity saturation curve is smoothed by 7-day moving averages.

Using embryo weights, 89 conception date were estimated. The dates of the first and last conceptions are 16 August 2019 and 25 December 2019, respectively. According to the saturation curve of cumulated conception data illustrating the dynamics (temporal trends) of conception, 93% of the conceptions detected could be dated to the interval between 16 August and 23 October, with only 7% taking place in the period afterwards. The saturation curve shows that the rate of gravid hinds exceeded 20% on 31 August (D_243_) and, rising steeply, reached 60% within 10 days, by 9 September (D_252_). Then, the rate of increase dropped. By 19 September (D_262_), the rate of gravid hinds was 80%. This 20-day period (between D_243_ and D_262_) was considered as the main period “Rut” (R), when 60% of the conceptions took place. The conception rate of this 20-day main period significantly differs from those of both the main periods BR (D_213-242_) and AR (D_263-302_) according to results of the *F* test (*F* = 35.725; *p* ≤ 0.001) (Figure 1). 

**Figure 2:**
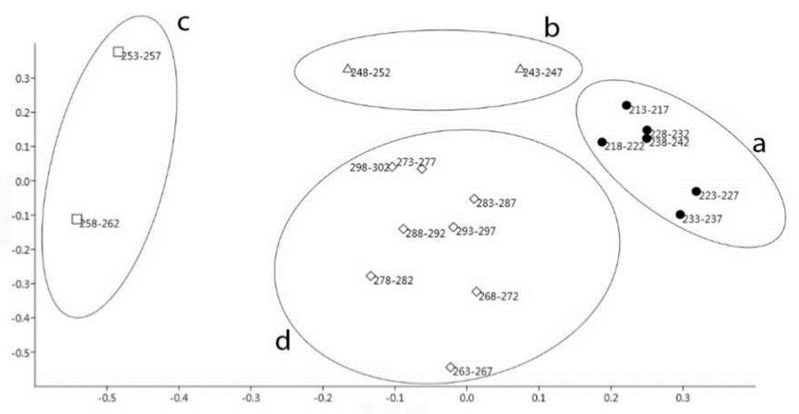
Grouping of activity data according to the results of PCoA (**a**—Before Rut (BR), 1 August–30 August; **b**—First Half of Rut (FHR) 31 August–9 September; **c**—Second Half of Rut (SHR) 10 September–19 September; **d**—After Rut (AR), 20 September–29 October).

The activity curve of stags was plotted against the days of the study period. Activity was expressed as a percentage of the maximum daily average activity of marked individuals (7565 m). The curve was smoothed by 7-day moving averages (Figure 1). The *F*-test was used to analyse the sections of the curve defined by the system used for the analysis of the conception data. Results indicated a significant difference between the activity data in the three main periods (BR, R, and AR) (*F* = 93.757; *p* ≤ 0.001). A shift may be observed between the peaks of conception and activity within the main period Rut. To analyse this shift, PCoA was performed for the daily activities of the 5-day periods starting with 1 August (Figure 2). 

**Figure 3:**
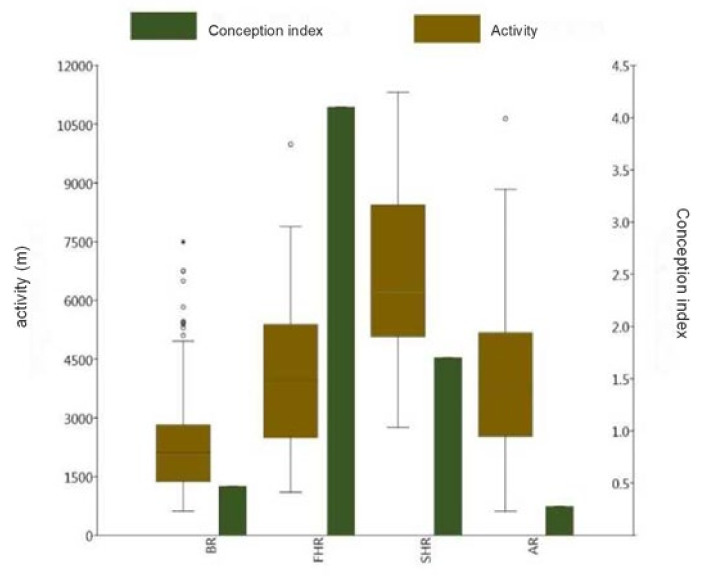
Activity and conception index values in the different study periods (“Before Rut” (BR), 1 August–30 August; First Half of Rut (FHR) 31 August–9 September; Second Half of Rut (SHR) 10 September–19 September; After Rut (AR), 20 September–29 October).

Regarding the 5-day sections defined according to the conception curve, the main periods BR (Figure 2a; D_213-242_) and AR (Figure 2d; D_263-302_) are clearly distinguishable both from each other and from the main period Rut. However, data in the main period Rut do not form a homogenous group, hence the division of the two periods, thus creating the main periods FHR (Figure 2b; D_243-252_) and SHR (Figure 2c; D_253-262_). In the next step, activity and conception index data were compared for the four main periods (Figure 3).

Average daily values for activity were calculated. A statistically significant difference between main periods was confirmed by One-way ANOVA (*F*_(3, 356)_ = 60.12, *p* ≤ 0.001). Tukey’s HSD indicated significant differences between five parings (BR↔FHR, BR↔SHR, BR↔AR, FHR↔SHR, and SHR↔AR), and averages in the FHR↔AR comparison did not differ (Table 1.).

The values of the conception index were created to facilitate the comparison of conception characteristics. Differences were analysed using the χ^2^ test. Statistical analysis verified significant differences in three pairings (BR↔FHR, FHR↔SHR, and FHR↔AR). Statistical differences could not be demonstrated for another three pairings (BR↔SHR, BR↔AR, and SHR↔AR) (Table 2.).

The average movement ranges in the quints of the main periods as calculated by the Kernel90 method were statistically different (One-way ANOVA, *F*_(3, 68)_ = 15.63, *p* ≤ 0.001). The largest movement range was found for SHR; it was significantly different from all other values. As for pairwise comparisons, the difference in the case of SHR↔BR was 11.2-fold, while in the case of SHR↔FHR, it was 4.3-fold and for SHR↔AR, it was 2.9-fold, respectively. Differences between the other main periods are as follows: BR↔AR: 3.9-fold; BR↔FHR: 2.6-fold; and FHR↔AR: 1.5-fold (Table 3.).

**Figure 4:**
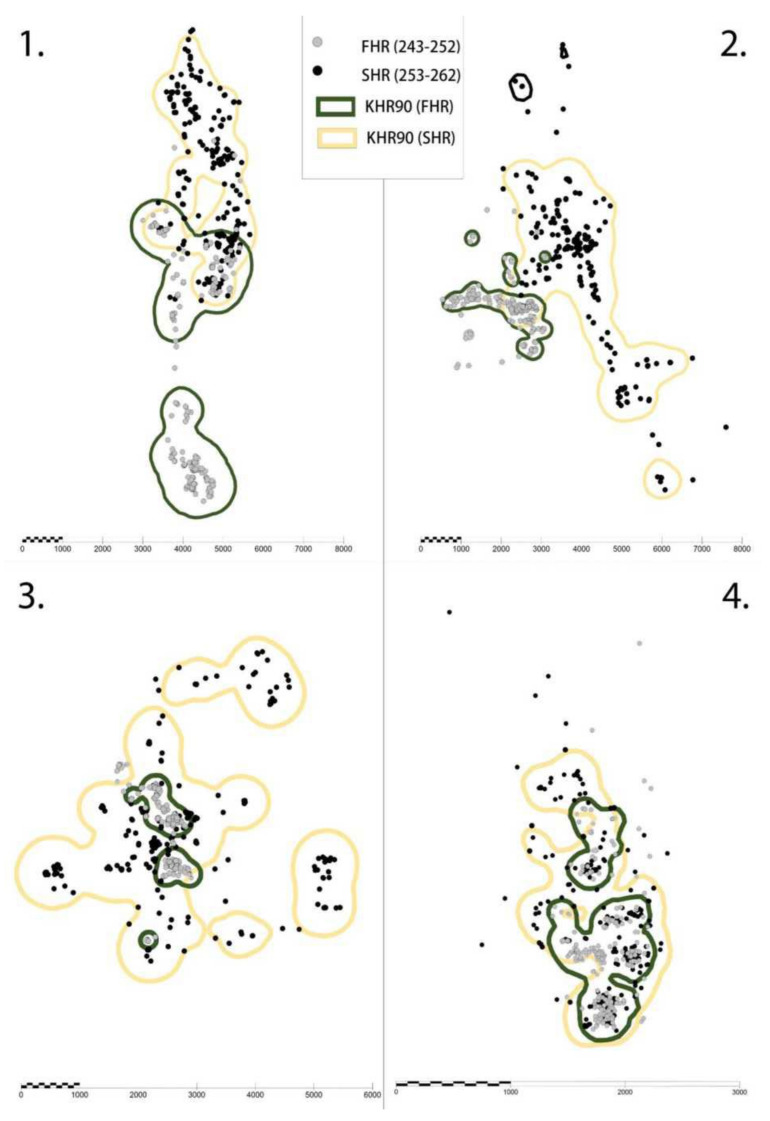
Detected positions of four marked red deer stags (**1**–**4**) and their movement range calculated by the Kernel method in the main periods First Half of Rut (FHR) and Second Half of Rut (SHR).

Relying on these results, we could prove that the four main periods were clearly distinguishable regarding both activity and conception characteristics and the activity of stags increased as the number of hinds on heat decreased. To prove this hypothesis, we analysed the spatial distribution of the detected positions of marked animals. Map visualisation confirms the change in movement range values. This suggests an increase in the range of movement and a decrease in the concentration of positions due to search behaviour for all four marked individuals. (Figure 4). 

## 4. Discussion

Based on our results, we can conclude that 80% of hinds conceived until 19 September, and 60% conceived between 31 August and 19 September. This interval (20 days) should be considered as the main rutting period in the studied year. Our results showed earlier and more concentrated rutting peaks than in other habitats [59]. The concentrated rutting period presumes a high proportion of prime-aged stags. However, young males can participate in the rut but do not have the same capacity to inseminate as many females as prime-aged males [60]. Examining the main rutting period, we demonstrated that the first half (D_243-252_) differed significantly from its second half (D_253-262_) regarding conception rates. In the first half (31 August and 9 September), the conception index was 4.1, i.e., 40% of hinds conceived during these 10 days. Early conception and the rapid increase in the rate of conceived hinds may result from high oestrus synchrony [25], which is particularly important for the subsequent survival of calves [61]. The high density of hinds in the study area may affect the competition between stags [27,62,63], as well as the age composition of stags and the roaring activity in the rutting territories, which can also affect the behaviour (oestrus) of females [64]. The behaviour of stags is known to change in the rutting season [30]. Age and dominance ranking may also influence it [65]. Our observations in the study area also demonstrated this fact. However, it is necessary to note that our findings for stags are age-specific for the middle-aged class due to the low number of marked individuals. In the case of younger or older individuals, different behavioural norms may occur.

During the main rutting period, marked individuals showed increased activity in the first half of September (compared to the period before the start of the rut). This behavioural phenomenon was expected as the marked individuals were prime-aged stags. Showing harem-holding strategy fits the conceptual model of Bowyer et al. until the first half of the rut. Female Defensibility is high. Suppose that the defensibility of hinds and the density of hinds in oestrus decreases; in that case, Polygynous Resource Territory is predicted [66].

However, the activity of stags within the rut season depended on the conception rate of hinds. We demonstrated a lower activity in the first half of the rut (FHR) characterised by a higher conception index than in the second half (SHR), where the conception index was lower. This difference in the activity of stags was induced by the decrease in the number of hinds still in oestrus, due to which the harem-herding behaviour of stags switched to searching behaviour. The same trends were observed by Jarnemo et al.; in their study, the number of hinds observed in the study area in September was constant; however, this was not true for the adult stags. Between 10 and 20 September, the number of stag observations increased rapidly and then started to fall [67]. These results are similar to the changes in activity observed by us.

Stags showing increased activity have a higher chance of meeting fertile hinds. The extended movement could demonstrate these searching behaviour ranges found in the second half of the rut (SHR), which showed a 4.3-fold increase compared to the first half of the rut in the case of KHR90. The process we observe can be considered a modified polygynous resource territory behaviour. We also proved that the searching behaviour and the related increased activity were maintained by stags as long as the number of hinds in oestrus justified it. Once cumulated conception had reached 80%, activity and movement range values started to drop. This indicates that the energy invested in reproduction was no longer affordable for stags, and survival was again a higher priority than passing their genetic material [68,69]. Activity values decreased to the level observed in the first half of the rut (FHR) but still exceeded, however, those measured before the rut (BR). 

The growing population of red deer and the increase in activities due to habitats disturbance raise several game management issues. Long-distance migration is a known influence on species conservation and management [70]. Movement between management units also limits effective deer management [71]. Recreational hunting and game management harvest are important tools for influencing the red deer population, but they can also affect red deer movement activity. [72]. Based on our results, in addition to the factors listed above that affect the red deer movement behaviour, the knowledge of the activity of stags during the rut should be considered in game management, particularly in smaller game management units.

## 5. Conclusions

We can conclude that the fertile period of red deer hinds during the mating season can significantly affected stag activity. At the beginning of the reproductive cycle, the high number of hinds on heat (oestrus synchrony) increased the activity of stags (harem herding) compared to the pre-rutting period. As the mating season progressed, the activity of stags increased more (searching behaviour), induced by the decreasing number of fertile hinds. Stags maintained this searching behaviour as long as the number of fertile hinds decreased drastically, stags reduced their activity, promoting activities to their own survival.

## Figures and Tables

**Table 1:** The result of analysis of average daily activity.

Average Daily Activity (Meter)		Tukey’s HSD Results									
				BR		FHR		SHR		AR	
	
Before Rut (BR)	2403	BR				* p ≤ 0.001		* p ≤ 0.001		* p ≤ 0.001	
	
First Half of Rut (FHR)	4870	FHR		7.379				* p ≤ 0.001		*p = 0.983*	
	
Second Half of Rut (SHR)	6638	SHR		18.55		9.123				* p ≤ 0.001	
	
After the Rut (AR)	3972	AR		10.4		0.5199		12.06			
	

Tukey’s Q below the diagonal, *p* above the diagonal, * significant difference.

**Table 2:** The result of analysis of conception index.

Conception Index		χ2 Test Results									
				BR		FHR		SHR		AR	
	
Before Rut (BR)	0.47	BR				*p ≤ 0.001		*p = 0.692*		*p = 0.664*	
	
First Half of Rut (FHR)	4.1	FHR		19.822				*p ≤ 0.001		*p = 0.307*	
	
Second Half of Rut (SHR)	1.7	SHR		0.356		15.264				*p ≤ 0.001	
	
After the Rut (AR)	0.28	AR		0.424		1.546		25.2			
	

χ2 below the diagonal, *p* above the diagonal, * significant difference.

**Table 3:** The result of analysis of average movement range.

Average Movement Range Kernel Home Range 90 (ha)		Tukey’s HSD Results									
				BR		FHR		SHR		AR	
	
Before Rut (BR)	67.7	BR				*p = 0.708*		* p ≤ 0.001		* p = 0.022	
	
First Half of Rut (FHR)	176.7	FHR		1.516				* p ≤ 0.001		*p = 0.802*	
	
Second Half of Rut (SHR)	757.4	SHR		9.597		6.598				* p ≤ 0.001	
	
After the Rut (AR)	265.8	AR		4.167		1.281		7.065			
	

Tukey’s Q below the diagonal, *p* above the diagonal, * significant difference.

## Data Availability

The datasets generated and analysed during the current study are not publicly available but are available from the corresponding author upon reasonable request.
